# Books and Pamphlets Received

**Published:** 1884-10-20

**Authors:** 


### BOOKS AND PAMPHLETS RECEIVED.

*Transactions of the Medical Society of the State of West Virginia.* Seventeenth Annual Session, held in Clarksburg, May 21 and 22, 1884.

We are indebted to the kindness of the Secretary, Dr. S. L. Jepson, of Wheeling, for a copy of the above work.

An address of welcome was made by Dr. D. P. Morgan, also by Gen. R. S. Northcut, Mayor.

The officers elect are:

President—Dr. Geo. Baird.

Vice Presidents—Dr. T. A. Harris, R. R. Foly, S. H. Austin.

Treasurer—Dr. Jno. A. Campbell.

Secretary—Dr. Samuel L. Jepson.

Board of Censors—J. H. Manown, L. C. Hunt, J. H. Brownfield, J. O. Wall, J. M. Bacock, W. J. Bland.

The following gentlemen presented papers to the Society: Drs. R. W. Hall, R. M. Baird, Fleming Howell, Geo. H. Rohe, W. H. Sharp, J. P. Miller, Jno. Fressell, W. K. Curtiss.

The next meeting was appointed for the third Wednesday of May next at Weston.

*A Compend of Organic and Medical Chemistry*, including Urinary Analysis and the examination of Water and Food, by Henry Leffmann, M.D., D.D.S., Professor of Chemistry and Metallurgy in the Pennsylvania College of Dental Surgery, and of Clinical Chemistry and Hygiene in the Philadelphia Polyclinic. Philadelphia: P. Blakiston, Son & Co.

The above is a compact and valuable little work on Chemistry, of 124 pages, presenting in a nutshell the facts and principles of organic and medical chemistry—an excellent work for the student.

*Leffel's House Plans.*—We are in receipt of a copy of this work, just published by James Leffel & Co., 110 Liberty st., New York. It is a volume of more than two hundred pages, containing upward of one hundred and fifty illustrations. It comprises elevations and plans, with descriptions, of forty houses costing from five hundred dollars to three thousand dollars, and adapted to families having good taste and moderate means. The book is printed on heavy paper of the best quality, with clear type and illustrations, and handsomely bound in cloth, with blue and gold cover.

*On the Diagnosis of Tumors of the Anterior Mediastinum*, by James C. Wilson, M.D., Philadelphia, Lecturer on Physical Diagnosis at the Jefferson Medical College, etc., etc. Read in the Section on Practice of Medicine and Materia Medica, of American Medical Association, May, 1884. Reprinted from the Journal of the American Medical Association, July 12, 1884.

*Preventable Blindness*, by Samuel Theobald, M.D., Chairman, Professor of Diseases of the Eye and Ear in the Baltimore Polyclinic and Post-Graduate Medical School; Surgeon to the Baltimore Eye, Ear and Throat Charity Hospital. Reprint from Transactions of the Medical and Chirurgical Faculty of Maryland, 1884.

*Gunshot Wounds of the Small Intestines*, by Charles T. Parkes, M.D., Professor of Anatomy in Rush Medical College, Chicago, Ill., being the Address of the Chairman of the Section on Surgery and Anatomy, read at the meeting of the American Medical Association, held in Washington, D. C., May, 1884.

*Explanation of the Pathology and Therapeutics of the Diseases of the Nerve Centres*, especially epilepsy. By J. M. Gaston, M.D., Professor of Surgery in the SOUTHERN MEDICAL COLLEGE, Atlanta, Ga. Reprint from Transactions of The Georgia Medical Association.

*Proceedings, Addresses and Discussions* of the Third Semi-annual Meeting of the Kentucky State Sanitary Council, held at Bardstown, Ky., March 26th and 27th, 1884, under the auspices of the State Board of Health.

